# Macrosomia and Hyperinsulinaemic Hypoglycaemia in Patients with Heterozygous Mutations in the *HNF4A* Gene

**DOI:** 10.1371/journal.pmed.0040118

**Published:** 2007-04-03

**Authors:** Ewan R Pearson, Sylvia F Boj, Anna M Steele, Timothy Barrett, Karen Stals, Julian P Shield, Sian Ellard, Jorge Ferrer, Andrew T Hattersley

**Affiliations:** 1 Peninsula Medical School, Exeter, United Kingdom; 2 Division of Medicine and Therapeutics, Ninewells Hospital and Medical School, University of Dundee, Dundee, United Kingdom; 3 Department of Endocrinology, Hospital Clinic de Barcelona, Barcelona, Spain; 4 Genomic Programming of Beta Cells Laboratory, Institut d'Investigacions Biomèdiques August Pi i Sunyer, Barcelona, Spain; 5 Institute of Child Health, Birmingham, United Kingdom; 6 Bristol Royal Hospital for Children, Bristol, United Kingdom; 7 University of Bristol, Bristol, United Kingdom; Lund University, Sweden

## Abstract

**Background:**

Macrosomia is associated with considerable neonatal and maternal morbidity. Factors that predict macrosomia are poorly understood. The increased rate of macrosomia in the offspring of pregnant women with diabetes and in congenital hyperinsulinaemia is mediated by increased foetal insulin secretion. We assessed the in utero and neonatal role of two key regulators of pancreatic insulin secretion by studying birthweight and the incidence of neonatal hypoglycaemia in patients with heterozygous mutations in the maturity-onset diabetes of the young (MODY) genes *HNF4A* (encoding HNF-4α) and *HNF1A/TCF1* (encoding HNF-1α), and the effect of pancreatic deletion of *Hnf4a* on foetal and neonatal insulin secretion in mice.

**Methods and Findings:**

We examined birthweight and hypoglycaemia in 108 patients from families with diabetes due to *HNF4A* mutations, and 134 patients from families with *HNF1A* mutations. Birthweight was increased by a median of 790 g in *HNF4A*-mutation carriers compared to non-mutation family members (*p* < 0.001); 56% (30/54) of *HNF4A*-mutation carriers were macrosomic compared with 13% (7/54) of non-mutation family members (*p* < 0.001). Transient hypoglycaemia was reported in 8/54 infants with heterozygous *HNF4A* mutations, but was reported in none of 54 non-mutation carriers (*p* = 0.003). There was documented hyperinsulinaemia in three cases. Birthweight and prevalence of neonatal hypoglycaemia were not increased in *HNF1A*-mutation carriers. Mice with pancreatic β-cell deletion of *Hnf4a* had hyperinsulinaemia in utero and hyperinsulinaemic hypoglycaemia at birth.

**Conclusions:**

*HNF4A* mutations are associated with a considerable increase in birthweight and macrosomia, and are a novel cause of neonatal hypoglycaemia. This study establishes a key role for *HNF4A* in determining foetal birthweight, and uncovers an unanticipated feature of the natural history of *HNF4A*-deficient diabetes, with hyperinsulinaemia at birth evolving to decreased insulin secretion and diabetes later in life.

## Abstract 2

*HNF4A* mutations were found to be associated with a considerable increase in birthweight and macrosomia, and were a cause of neonatal hypoglycaemia.

## Editors' Summary

**Background.:**

MODY, or maturity-onset diabetes of the young, is a particular subtype of diabetes; only a few percent of people with diabetes are thought to have this subtype. The condition comes about as a result of a mutation in one of six genes. Generally, people with MODY have high glucose (sugar) levels in the blood, and the typical symptoms of diabetes, such as increased thirst and urination, typically develop when the person is below the age of 25 y. Two of the genes that are known to cause MODY are mutant forms of *HNF4A* and *HNF1A.* The proteins that are encoded by these two genes control insulin levels produced by the pancreas; when these genes are mutated, not enough insulin is produced. Without enough insulin to control blood sugar, levels rise, leading to the symptoms of diabetes. However, MODY can be managed by many of the same interventions as other types of diabetes, such as diet, exercise, drug treatments, and insulin injections.

**Why Was This Study Done?:**

Although the evidence shows that individuals who carry mutations in *HNF4A* and *HNF1A* do not produce enough insulin and therefore have higher glucose levels in their blood, there were some tantalizing suggestions from mouse experiments that this might not be the whole story. Specifically, the researchers suspected that during embryonic development, mutations in *HNF4A* or *HNF1A* might actually cause higher insulin levels. Too much insulin during development of a fetus is known to cause it to gain weight, resulting in a baby that is larger than the average size for its age. Larger babies are risky for both the baby and the mother. The researchers doing this study wanted to understand more precisely what the links were between the forms of MODY caused by *HNF4A* and *HNF1A* mutations, and birth-weight and blood-sugar levels.

**What Did the Researchers Do and Find?:**

In this study, the researchers examined 15 families in which some family members had MODY caused by a mutation in *HNF4A.* They compared the birthweight for family members carrying the mutation (54 people) against the birthweight for those who did not (54 people). A similar comparison was done for 38 families in which some members had a different form of MODY, this time caused by a mutation in *HNF1A.* The results showed that the birthweight of family members who carried a mutation in *HNF4A* was, on average, 790 g higher than the birthweight of family members who didn't carry the mutation. Low blood-sugar levels at birth were also more common in people carrying the *HNF4A* mutation as compared to people who did not. However, the *HNF1A* mutation did not seem to be associated with greater birthweight or low blood-sugar levels at birth. Finally, in order to understand these findings further, the researchers created embryonic mice carrying mutations in the mouse equivalent of *HNF4A.* These embryos produced more insulin than normal mouse embryos and, after birth, were more likely to have low blood-sugar levels.

**What Do These Findings Mean?:**

These findings show that there is a link between mutations in *HNF4A,* but not in *HNF1A,* and increased birthweight. The increase found in this study is quite substantial (a median weight of 4,660 g in the affected babies; a birthweight of more than 4,000 g is generally considered large). The results suggest that in human embryos with a mutated form of *HNF4A,* too much insulin is produced during development, causing faster growth and a higher chance of the baby being born with low blood-sugar levels. This is an unexpected finding, because later in life the *HNF4A* mutation causes lower insulin levels. Therefore, the biochemical pathways causing this type of MODY seem to be quite complicated, and further research will need to be done to fully understand them. Crucially, the research also suggests that pregnant women carrying *HNF4A* mutations should be closely followed to check their baby's growth and minimize the chance of complications. Doctors and families should also consider doing a genetic test for *HNF4A* if a baby has low blood-sugar levels and if there is a family history of diabetes; this would increase the chance of diagnosing MODY early.

**Additional Information.:**

Please access these Web sites via the online version of this summary at http://dx.doi.org/10.1371/journal.pmed 0040118.

- In a related Perspective in *PLoS Medicine,* Benjamin Glaser discusses causes of type 2 diabetes mellitus in the context of this study's findings
- The US National Institute of Diabetes and Digestive and Kidney Diseases has pages of information on different types of diabetes
- Wikipedia has an entry on Maturity Onset Diabetes of the Young (MODY) (note that Wikipedia is an internet encyclopedia that anyone can edit)
- Diabetes Research Department, Peninsula Medical School, Exeter, UK provides information for patients and doctors on genetic types of diabetes; the website is maintained by the research group carrying out this study
- Information from the Centers for Disease Control and Prevention on diabetes and pregnancy

## Introduction

Macrosomia is associated with considerable foetal and maternal morbidity [1]. Factors that predict macrosomia are still poorly understood [2]. In humans, foetal insulin secretion is one of the key determinants of foetal growth, acting mainly in the third trimester when the weight of the foetus increases greatly. This is seen in pregnant women with diabetes when foetal sensing of maternal hyperglycemia drives insulin secretion, insulin-mediated growth, and subsequent macrosomia. In addition to such environmental factors, mutations in the genes involved in insulin secretion are also known to affect birthweight. Mutations that cause hyperinsulinaemic hypoglycaemia of infancy [3–10] are associated with increased birthweight. Conversely, genes in which mutations cause neonatal diabetes [11,12] and some forms of maturity-onset diabetes of the young (MODY) [13,14] are associated with decreased birthweight.

The transcription factors hepatocyte nuclear factor-4α (encoded by the *HNF4A* gene), and hepatocyte nuclear factor-1α (encoded by *HNF1A,* approved gene name *TCF1*) play a key role in the regulation of pancreatic insulin secretion. *HNF4A* and *HNF1A* mutations cause monogenic diabetes (MODY 1 and MODY3, respectively) due to decreased insulin secretion [15,16] and are key parts of an important β-cell network [17,18]. In the pancreas, *HNF1A* and *HNF4A* form part of a common transcriptional network, which has been proposed as an explanation of the shared pancreatic phenotype seen in patients with mutations in these genes [18]. Variants in the *HNF4A* pancreatic promoter have also been associated with Type 2 diabetes [19,20].

A recent study reported mildly reduced blood glucose and increased insulin levels in adult β-cell *Hnf4a*–deficient mice [21]. As a result of this animal study, we hypothesized that mutations in the human gene *HNF4A* might increase foetal insulin secretion and birthweight, and cause neonatal hyperinsulinaemia and hypoglycaemia. We therefore studied birthweight and reported hypoglycaemia in *HNF4A*-mutation carriers and unaffected family members. As a comparison, we also studied the families of patients with mutations in the closely associated pancreatic transcription factor *HNF1A.* To investigate the mechanism of neonatal hypoglycaemia and increased birthweight, we also studied foetal and neonatal mice lacking both copies of *Hnf4a* in the pancreas.

## Methods

### Birthweight and Reported Hypoglycaemia in *Hnf4a*-Mutation Carriers

One hundred and eight members (54 mutation carriers) of 15 families who had been found to have MODY due to an *HNF4A* mutation, were contacted. This group included 13 families where the mutation had previously been identified as well as two families, found after screening for *HNF4A,* where there was both neonatal hypoglycaemia and diabetes in a family member (see below). Where mutation status of an individual within a family had not previously been determined, DNA was extracted from a buccal sample. *HNF4A* was amplified and sequenced as previously described [22]. Birthweight and gestational age were primarily obtained by maternal recall. Birth centiles and weight were corrected to 40 wk of gestation and for male sex, according to UK 1990 reference curves [23].

In assessment of neonatal hypoglycaemia, we report here on three patients from two families described above in whom *HNF4A* mutations had been identified due to coexistent familial diabetes and neonatal hypoglycaemia. In addition, we contacted 101 unselected members (48 mutation carriers) of the 13 families described above who had been found previously to have MODY due to an *HNF4A* mutation. In babies born to mothers with diabetes during pregnancy, hypoglycaemia that did not require intravenous glucose and lasted for less than 24 h was not considered exceptional. Any other reported incidence of hypoglycaemia at birth was followed up by case-note review. An episode of hypoglycaemia was established only if venous plasma glucose of less than 2.5 mmol/l was documented. All investigations for hyperinsulinaemic hypoglycaemia were done by the referring clinician at the time of diagnosis using their local laboratory.

### *HNF4A* Mutations in Families with both Diabetes and Hypoglycaemia

After our initial observations in families with known *HNF4A* mutations, we went on to sequence the *HNF4A* gene in the probands of five further families who had been referred to the Exeter Molecular Genetics Laboratory with hyperinsulinaemic hypoglycaemia, and who had at least one first-degree relative with diabetes. No monogenic cause had been found for the hypoglycaemia or diabetes in these families: three of the hypoglycaemic probands had been sequenced for activating *GCK* and/or *KCNJ11* mutations and, in one family, members with diabetes had been sequenced for *HNF1A* mutations.

### Birthweight and Reported Hypoglycaemia in *HNF1A*-Mutation Carriers

One hundred and thirty-four members (85 mutation carriers) from 38 families with known MODY due to an *HNF1A* mutation were contacted. Reported birthweight and hypoglycaemia were recorded in the same way as for *HNF4A*-mutation carriers.

### Transgenic Mice

β-cell–specific *Hnf4a* mutations (β-*Hnf4a-*KO) were generated by crossing *Hnf4a* ^LoxP/LoxP^ mice (Jackson Laboratory, http://www.jax.org) [24] with InsPr-Cre mice, a transgenic line expressing Cre recombinase driven by the rat insulin 2 promoter [25]. Both lines were bred on a C57BL/6J genetic background. An almost complete efficiency of recombination of *Hnf4a* LoxP alleles in β-cells was verified by: (1) real-time PCR quantitation in RNA from isolated islets showing an 80% reduction of *Hnf4a* mRNA relative to control mice (Figure S1); (2) absent Hnf-4α staining in >90% β-cells on immunofluroescence analysis (unpublished data); and (3) recombination of a Rosa26LoxP-Stop-LoxP-Lacz allele in >90% of β-cells when crossed with InsPr-Cre (unpublished data). Blood insulin and glucose were obtained after decapitation of timed embryos or neonatal mice (15–20 mice per genotype and stage). Glucose was measured with a glucose meter (Accu-Chek, Roche [http://www.roche.com]). Plasma insulin levels were determined by ELISA (Mercodia, http://www.mercodia.com). Genotyping was performed by PCR analysis using genomic DNA isolated from the tail tips of embryos and newborn mice.

### RNA Analysis

Isolated islets from 6–8-wk-old β-*HNF4a-*KO mice and control *Hnf4a* ^LoxP/LoxP^ littermates were used for RNA extraction with Trizol reagent (Invitrogen, http://www.invitrogen.com). RNA integrity was verified with the 2100 Bioanalyzer (Agilent Technologies, http://www.agilent.com) prior to reverse transcription and real-time PCR quantitation as described [26]. Oligonucleotide sequences are available upon request.

### Statistical Analysis

Owing to the non-normal distribution of birthweight data, non-parametric analysis was used. Median centiles and birthweights were compared using the Mann-Whitney U-test. For the discordant sibling analysis, the median birthweight corrected for sex and gestation for all mutation carriers and non-mutation carriers within a sibship were compared using the Wilcoxon signed rank test. Hypoglycaemia and macrosomia categorical data were compared using Fisher's Exact test. Data from the mouse studies are presented as mean ± standard error of the mean, and were compared by the two-tailed Student's *t*-test.

### Ethical Approval

This study was approved by the North and East Devon Regional Ethics Committee, UK and the Animal Ethics Committee of the University of Barcelona School of Medicine, Spain. All patients or carers gave informed consent.

## Results

### Birthweight in *HNF4A*-Mutation Carriers

**Figure 1:**
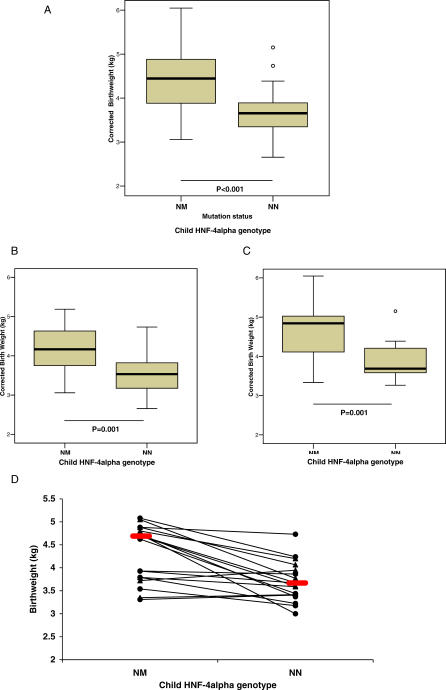
Birthweight (Adjusted for Sex and Gestational Age) according to Foetal Genotype (A) All offspring; (B) *HNF4A* mutation inherited from the father; (C) *HNF4A* mutation inherited from the mother, and (D) siblings discordant for *HNF4A* genotype. Foetal genotype: NM, heterozygous *HNF4A* mutation; NN, normal *HNF4A.* For (A) to (C), bars represent median, the box represents interquartile range, and the whiskers represent the range, with outliers shown as circles. Comparing NM birthweight with NN birthweight by Mann-Whitney U-test: *p* < 0.001 for (A) all offspring; *p* = 0.001 for (B) father affected and for (C) mother affected. In (D), those pairs where the father has the mutation are shown as filled circles; those pairs where the mother has the mutation are shown as filled triangles. The red bars represent median birthweight.

The characteristics of all the *HNF4A*-mutation carriers and their unaffected family members are shown in Table 1. The median birthweight of the *HNF4A*-mutation carriers was the 96th centile (interquartile range 75–100) compared with the 58th centile (interquartile range 33–76) in unaffected family members, giving a difference in corrected median birthweight of 790 g, *p* < 0.001 (Figure 1A). A difference in birthweight was seen both when the mutation was inherited from the father, *p* < 0.001 (Figure 1B) or from the mother, *p* < 0.001 (Figure 1C). There was no effect of offspring genotype on gestational age at delivery (*p* = 0.29). The influence of *HNF4A* genotype on birthweight remained significant if the individuals from the two families referred with hyperinsulinaemia were excluded (*p* < 0.001). Nineteen mothers who were mutation carriers and one mother who was not a mutation carrier had diabetes during pregnancy; 11 of the 20 offspring from these pregnancies were mutation carriers. Finally, to allow for any impact of maternal hyperglycaemia, corrected median birthweight was compared in 18 sibling pairs discordant for the presence of the *HNF4A* mutation. The median birthweight of the mutation-carrying siblings was 4,660 g compared with a median birthweight of the non-mutation-carrying siblings of 3,640 g, *p* = 0.001 (Figure 1D).

**Table 1:**
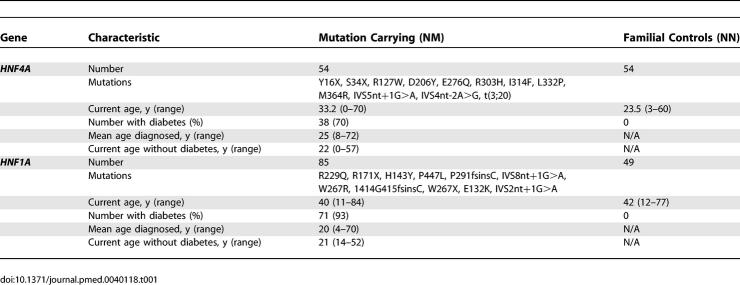
Characteristics of Patients

Macrosomia, defined as a birthweight of more than 4,000 g, was present in 56% of *HNF4A*-mutation carriers but in only 13% of non-mutation carriers (*p* < 0.001). The prevalence of macrosomia was 64% if the *HNF4A* mutation was inherited from the mother and 46% if the *HNF4A* mutation was inherited from the father. In contrast in an unaffected foetus, the equivalent rates were 25% with an affected mother (*p* = 0.07), and 6% with an affected father (*p* = 0.003). Macrosomia is associated with increased foetal and maternal morbidity, and this was seen in some of the patients. The deliveries of the two siblings from family 1,023 were both complicated by severe shoulder dystocia, with 1,023–1 developing an Erb's palsy. The prevalence of extreme macrosomia, defined as a birthweight of >5,000 g, which is associated with increased neonatal mortality [27], was 15% (four neonates) in *HNF4A*-mutation carriers with an affected mother and 7% (two neonates) in *HNF4A*-mutation carriers with an affected father. No non-mutation carriers had extreme macrosomia.

### Neonatal Hyperinsulinaemic Hypoglycaemia in *HNF4A*-Mutation Carriers

**Figure 2:**
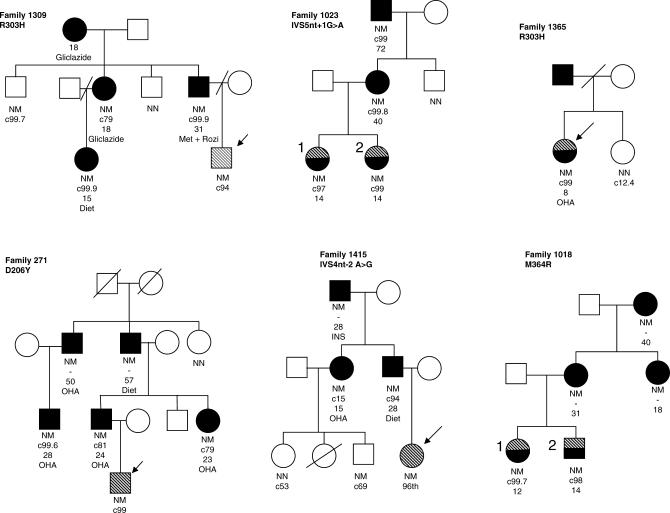
Pedigrees of Families with Hypoglycaemia Patients with hypoglycaemia are shaded with bold diagonal stripes; patients who had hypoglycaemia but have progressed onto diabetes are shaded with half black/half diagonal stripes; and patients with diabetes are coloured black. Probands are indicated with an arrow. Where available, below each symbol is recorded genotype (NM, *HNF4A*-mutation carrier; NN, unaffected), birth centile adjusted for sex and gestation, age at which diabetes developed, and the patient's treatment.

Transient neonatal hypoglycaemia is a feature of some *HNF4A*-mutation carriers. Two out of five families referred to Exeter, with hypoglycaemia and a first-degree family member with diabetes, were shown to have novel *HNF4A* mutations (M364R, IVS4nt-2A>G). Three out of the six mutation carriers in these families had documented neonatal hypoglycaemia (Figure 2; Table 2). In addition, five out of 48 *HNF4A*-mutation carriers in families previously identified with MODY had hypoglycaemia at or soon after birth lasting > 24 h and requiring treatment (Table 2). So, overall, eight out of 54 mutation carriers had neonatal hypoglycaemia compared to none of the 54 non-mutation carriers (*p* = 0.003). If the patients from the two additional families are excluded, there is still a significant excess of hypoglycaemia in the mutation carriers (*p* = 0.02).

**Table 2:**
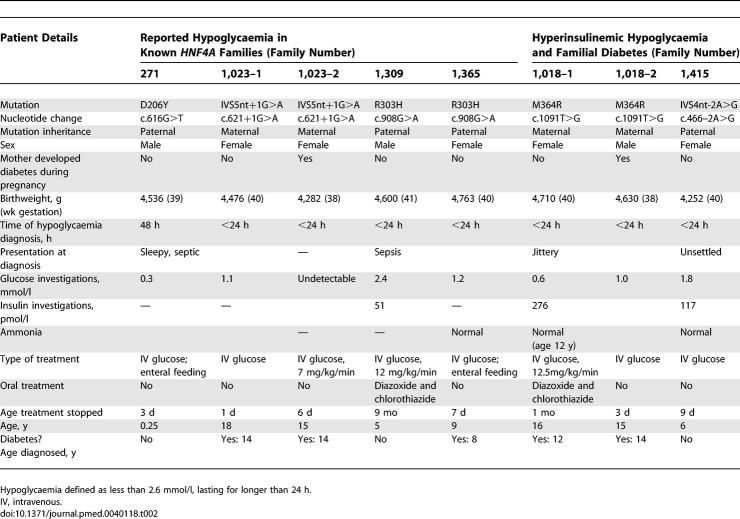
Clinical Features of Patients with *HNF4A* Mutations and Documented Hypoglycaemia

The clinical features of the eight cases with established hypoglycaemia during infancy associated with *HNF4A* mutations are shown in Table 2. Their pedigrees (Figure 2) show that hypoglycaemia was often described only in a single family member with other carriers of the same *HNF4A* mutation presenting with diabetes. The prevalence of hypoglycaemia was similar if the mutation was inherited from the father (four out of 27 patients) or from the mother (four out of 27 patients), suggesting that persisting neonatal hypoglycaemia is independent of maternal glycaemia in pregnancy. In six patients, the hypoglycaemia was treated with intravenous glucose and enteral feeding, but two patients (1,018–1 and 1,309) required treatment with diazoxide and chlorothiazide for 1 and 6 mo, respectively. In both cases, there was documented inappropriate hyperinsulinaemia in the presence of hypoglycaemia (Table 2).

### β-Cell Deletion of *Hnf4a* in Mice

**Figure 3:**
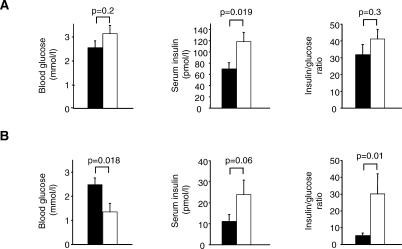
Deletion of *Hnf4a* in β-Cells Results in Hyperinsulinaemia and Hypoglycaemia in Mice (A) Blood glucose, plasma insulin, and insulin/glucose ratio of E18.5–E20 embryos. (B) Blood glucose levels, plasma insulin levels, and insulin/glucose ratio of newborn mice. Data from β-*Hnf4a-*KO and controls is shown in white and black bars, respectively. Values are mean ± standard error of the mean.

To directly test whether *Hnf4a* deficiency affects insulin secretion in utero, mice with β-cell–specific *Hnf4a* deletion (β-*Hnf4a*-KO) were examined during late gestation (E18.5–E20). Late-gestation β-*Hnf4a*-KO embryos exhibited significantly elevated insulin concentrations (118.14 ± 16.62 pmol/l versus 69.75 ± 11.03 pmol/l in β-*Hnf4a*-KO versus controls, *p* = 0.019) (Figure 3A). Glucose concentrations, which are regulated by the mother, were similar in both groups (Figure 3A). Birthweight, which in mice does not exhibit insulin dependence as in humans [28], did not differ in β-*Hnf4a*-KO mice. During the neonatal period, blood glucose values were low relative to control littermates (1.35 ± 0.35 mmol/l versus 2.50 ± 0.28 mmol/l, respectively, *p* = 0.018) (Figure 3B). Blood glucose levels below 1.1 mmol/l were observed at least seven times more frequently in neonatal β-*Hnf4a*-KO neonatal mice than in controls (8/15 versus 2/28, respectively, *p* < 0.001). This hypoglycaemia was due to increased insulin secretion, as the insulin/glucose ratio values in β-*Hnf4a*-KO versus control mice were 29.8 ± 12.0 versus 5.6 ± 1.5 (*p* = 0.01), while insulin values were 23.9 ± 6.9 pmol/l versus 11.1 ± 3.3 pmol/l (*p* = 0.053), respectively (Figure 3B). The expression of genes causing human hyperinsulinaemic hypoglycaemia, namely *Kcnj11* (encoding Kir6.2), *Abcc8* (encoding Sur1), *Schad, Gck,* and *Glud1,* was unaltered in *Hnf4a*-deficient islets (Figure S1). In summary, these findings indicate that *Hnf4a* deficiency causes hyperinsulinism during foetal and neonatal life, supporting the suggestion that this is the underlying cause of macrosomia and hypoglycaemia in *HNF4A*-mutation carriers.

### Birthweight and Hypoglycaemia in *HNF1A*-Mutation Carriers

**Figure 4:**
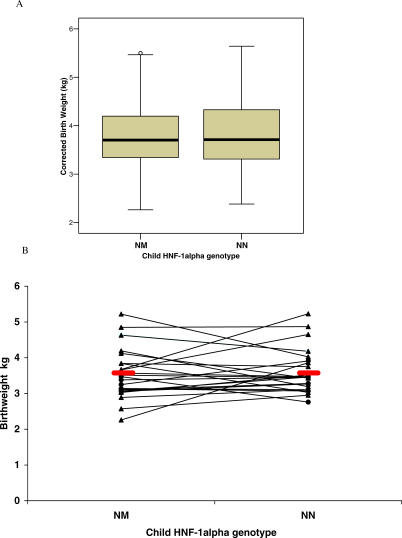
Birthweight in *HNF1A*-Mutation Carriers (A) Median centile (adjusted for sex and gestation) in mutation carriers (NM) and non-mutation carriers (NN) (*p* = 0.86). Error bars show interquartile range. (B) Discordant sib-pair analysis. Those pairs where the father has the mutation are shown as filled circles; those pairs where the mother has the mutation are shown as filled triangles. Median birthweight: NM 3,490 g; NN 3460 g (*p* = 0.91).

The characteristics of the *HNF1A*-mutation carriers and their unaffected family members are shown in Table 1. Unlike *HNF4A,* mutations in *HNF1A* are not associated with an increased birthweight (Figure 4A), with a median difference of 10 g (*p* = 0.86) and a mean difference in the analysis of 24 discordant sibling-pairs (Figure 4B) of 3g (*p* = 0.91). Only one *HNF1A*-mutation carrier had neonatal hypoglycaemia requiring intravenous glucose and persisting for longer than 24 h; however, his mother had diabetes during pregnancy, and he required less than 48 h of this treatment. Hypoglycaemia was more common in *HNF4A*-mutation carriers (eight out of 54) than in *HNF1A*-mutation carriers (one out of 77), *p* = 0.004.

## Discussion

We have shown that increased birthweight and macrosomia are common features of patients with *HNF4A* mutations and, in addition, that some individuals with *HNF4A* have neonatal hypoglycaemia. Although in young adults the same genetic defect results in diabetes due to reduced insulin secretion [15,22], we have shown that the mechanism for the phenotype in newborns is likely to be increased insulin secretion in utero and in the neonatal period. This is supported by hyperinsulinaemia in some affected infants with *HNF4A* mutations, and studies in mice with β-cell deletion of *Hnf4a* clearly show hyperinsulinaemia in utero and hyperinsulinaemic hypoglycaemia in the early neonatal period.

*HNF4A*-mutation carriers are, on average, 790 g heavier than their family members who do not carry the mutation, and 56% are born with macrosomia (>4,000 g). The increase in birthweight is similar to that seen in the offspring of patients with maternal diabetes which is the commonest recognized cause of macrosomia. Forty-six percent of children with an *HNF4A* mutation born to affected fathers had macrosomia. This is a clear example that macrosomia may result from foetal genetics as well as from the maternal intra-uterine environment. Consideration of this should be taken into account when determining macrosomia risk, and we recommend that, in addition to maternal diabetes, a history of young-onset non-insulin-requiring paternal diabetes should prompt assessment of foetal size.

We have described eight patients with *HNF4A* mutations who had one or more episodes of hypoglycaemia in the neonatal period; there was hyperinsulinaemia in all three patients who were tested. Five patients required treatment with intravenous glucose only, with resolution within 1 mo; this finding was consistent with a transient hyperinsulinaemia. Two patients had more persistent hypoglycaemia which responded well to treatment with diazoxide and chlorothiazide and subsequently resolved. Therefore, the loss of *HNF4A* function causes a relatively mild form of hyperinsulinaemic hypoglycaemia that is transient and diazoxide-responsive. Transient hypoglycaemia is often not investigated and as a result is understudied. We propose that neonates presenting with hypoglycaemia who have a father with diabetes, or a mother with young-onset non-insulin-requiring diabetes, should be screened for *HNF4A* mutations. However, three out of the five unselected *HNF4A*-mutation carriers with neonatal hypoglycaemia presented before their respective parent developed diabetes. Therefore, we also suggest that *HNF4A* mutations should be considered in any child with persistent hypoglycaemia (>24 h).

We encountered two problems resulting from the retrospective nature of this study. Firstly, hospital records were not readily available so birthweight and gestational age were ascertained by parental recall in the majority of cases. However, all this data collection was done blind to genotype and therefore any error should apply to both offspring groups. Secondly, the hypoglycaemia was often not well investigated at the time of presentation, presumably because of its transient nature. Hence, hyperinsulinaemia at birth was looked for only in three out of the eight patients presenting with hypoglycaemia, and other causes of hyperinsulinaemia were not excluded. It also means that we are uncertain whether the 46 patients with *HNF4A* mutations in whom hypoglycaemia was not described had undetected hypoglycaemia or were not hypoglycaemic. A prospective study of neonates born to *HNF4A*-mutation carriers is required for a complete assessment of the hyperinsulinaemic hypoglycaemia seen in these patients.

The increased birthweight and risk of macrosomia in *HNF4A*-mutation carriers is likely to be secondary to foetal hyperinsulinaemia. Although no measures of foetal insulin or cord insulin were available to confirm this mechanism in humans, two lines of evidence support it. Firstly, in humans, we have documented hyperinsulinaemia soon after birth in the three patients in whom it was tested, and hypoglycaemia in eight. Secondly, we have shown that mice lacking pancreatic *Hnf4a* have increased insulin concentrations in utero, and hyperinsulinaemic hypoglycaemia as newborns.

The increased birthweight and neonatal hypoglycaemia in *HNF4A-*mutation carriers seems paradoxical for a gene that is associated with a β-cell–deficient form of young-onset diabetes [15,22], particularly as the decreased β-cell function has been explained by decreased expression of pancreatic β-cell genes involved in glucose metabolism [29,30]. It is in contrast to other monogenic causes of diabetes—for example, *GCK* [13], *IPF1* [31], *HNF1B* [14], or activating *KCNJ11* [32,33] and *ABCC8* mutations [12,34] where birthweight is reduced. In these cases, the low insulin secretion that causes diabetes later is associated with decreased insulin-mediated foetal growth. In *HNF4A*-mutation carriers, in contrast, there would need to be a switch from increased insulin secretion in utero and neonatal life to decreased insulin secretion in later life. The closest example of this is patients with hyperinsulinaemia of infancy due to recessive and dominant mutations in K_ATP_ channel subunits, who have a high rate of diabetes at long-term follow-up even when they do not receive pancreatic surgery [8,35,36]. It has been postulated that diabetes in SUR1-deficient patients reflects increased apoptosis, in addition to abnormal regulation of secretion due to lack of K_ATP_ channels [37]. Compared to SUR1 deficiency, HNF-4α deficiency results in less severe hyperinsulinism, yet gives rise to a more highly penetrant and severe diabetic phenotype. It is interesting, however, that of the eight patients who developed established hypoglycaemia at birth, five developed diabetes by the age of 14 y (mean age 12.4 y), suggesting a possible earlier progression to diabetes in this group.

Two recent studies surprisingly showed that β-cell *Hnf4a* deficiency does not cause diabetes in mice [21,38]. One study paradoxically reported mildly reduced blood glucose and increased insulin levels in adult β-cell *Hnf4a*-deficient mice, and ascribed this to diminished expression of *Kcnj11* encoding the K_ATP_ channel subunit Kir6.2 [21]. Another study failed to confirm abnormal blood glucose and insulin levels, and reported normal *Kcnj11* expression [38]. This discrepancy, together with the unexpected failure to develop hypoinsulinaemic diabetes, led us to question whether hyperinsulinaemia was an important feature of *Hnf4a* deficiency. In the current study, we have shown that, in parallel with the human findings, *Hnf4a*–deficient mice exhibit hyperinsulinaemia in the foetal and neonatal stage, as well as overt neonatal hypoglycaemia as opposed to only mildly reduced glucose at later ages as recently reported [21]. Importantly, our studies showed no abnormal expression of *Kcnj11*. While discrepancies in phenotype might be explained by small differences in genetic background, the current data suggest that the hyperinsulinaemic phenotype in *Hnf4a* deficiency is not related to K_ATP_ channel expression.

Further studies will need to address how Hnf-4α-dependent transcription in β-cells is linked to the dual phenotype reported here. Large-scale profiling shows that Hnf-4α-deficient β-cells exhibit abnormal expression of more than 10% of all islet genes (unpublished observations), many of which need to be examined as plausible candidates for the *HNF4A*-deficient hypersecretory phenotype. It is tempting to hypothesize that the initial defect that causes β-cell hypersecretion might eventually lead to β-cell exhaustion and diabetes, in analogy to what is observed in some patients with SUR1 mutations as described above [8,35,36]. However, the broad transcriptional phenotype of *Hnf4a-*deficient mice offers an alternative potential explanation, whereby one HNF-4α-dependent gene-expression defect causes hypersecretion early in life, while a separate gene-expression defect is responsible for the development of severe β-cell failure several years after birth.

The birthweight and incidence of hypoglycaemia in heterozygous *HNF1A*-mutation carriers were not different from their unaffected family members. This finding suggests that foetal insulin secretion is not increased in *HNF1A*-mutation carriers. Previous data had supported a common phenotype of *HNF1A-* and *HNF4A*-mutation carriers, due to a regulatory transcription factor circuit in the β-cell with positive feedback on expression between HNF-1α and HNF-4α [18,22,39,40]. Our findings suggest that, at least in foetal life, there are clearly independent functions of these two transcription factors in the foetal β-cell. Our data therefore suggest that, in humans, the proposed transcription-factor network is not critically required in foetal development and early post-natal life.

**Box 1. Practical Clinical Points for Diagnosis and Management of Patients with *HNF4A* Mutations**

**Management of pregnancy in families known to have diabetes due to *HNF4A* mutations**

Serial antenatal scans should be performed in any pregnancy in which the father or mother is a mutation carrier and early induction of labour is considered. This is true when the mother does not have diabetes, but particularly applies when the mother has diabetes or impaired glucose tolerance in pregnancy.

All offspring of pregnancies where the father or mother is an *HNF4A*-mutation carrier should be tested for neonatal hypoglycaemia at birth and also 24 h after birth.

**Diagnosing MODY**

In families where there is autosomal dominant inheritance of young-onset diabetes with features consistent with a diagnosis of MODY, details of birthweight and neonatal hypoglycaemia should be specifically asked for.

When macrosomia or neonatal hypoglycaemia (>24 h) is described, *HNF4A* should be sequenced before other genes when performing diagnostic genetic testing.

**Diagnosing and managing neonatal hypoglycaemia**

*HNF4A* should be sequenced in children with neonatal hypoglycaemia, particularly if the hypoglycaemia is relatively mild or transient, or if a family member (parent or grandparent) has young-onset diabetes (<35 y).

In patients diagnosed as having *HNF4A,* resolution of symptoms should be expected in the first year, but diabetes should be expected to develop in adolescence or in early adulthood and should be screened for annually after the age of 10 y.

**Investigating macrosomia**

*HNF4A* should be sequenced as part of an investigation of unexplained macrosomia, particularly when the macrosomia is extreme, is accompanied by hypoglycaemia, or there is a family history of early-onset diabetes.

To conclude, we have shown that heterozygous *HNF4A* mutations are associated with a 790 g increase in birthweight, on average, and considerable risk of macrosomia. The increased birthweight is probably mediated by increased foetal insulin secretion and, in some cases, is associated with transient neonatal hyperinsulinaemia. Because *HNF4A* deficiency is also known to cause hypoinsulinaemic diabetes, this study shows for the first time that HNF-4α has dual opposing roles in the β-cell during different periods of life. This study also has important implications for clinical practice (see Box 1). Firstly, pregnancies where a parent is known to have an *HNF4A* mutation should be monitored closely during pregnancy and the immediate post-natal period to minimize complications of macrosomia and neonatal hypoglycaemia. Secondly, neonates with transient or persistent hypoglycaemia and/or macrosomia and a family history of young-onset diabetes should be considered for *HNF4A* molecular genetic testing. Thirdly, since the foetal genotype has a considerable impact on determining birthweight, in addition to maternal factors, paternal factors (including history of diabetes) should be considered when assessing macrosomia risk.

## Supporting Information

Alternative Language Abstract S1 Translation into Spanish Prepared by the Authors (29 KB DOC)

Figure S1 mRNA Levels of Genes Known to Cause Human Hyperinsulinism in Control and β-*Hnf4a-*KO Mice Data are mean ± standard error of the mean from three different control and mutant mice, except for *Kcnj11* where six control and six mutant mice were analysed.
(28 KB PPT)

### Accession Numbers

The GenBank (http://www.ncbi.nlm.nih.gov/Genbank) identification numbers for the genes and gene products discussed in this paper are *HNF1A/TCF1* (NM_000545.3) and *HNF4A* (NM_000457.3).

## Abbreviation

MODY: maturity-onset diabetes of the young
