# Supplementary material for: Macrosomia and Hyperinsulinaemic Hypoglycaemia in Patients with Heterozygous Mutations in the HNF4A Gene
Source: PLoS Med. 2007 Apr 3;4(4):e118. doi: 10.1371/journal.pmed.0040118 (PMC1845156; doi:10.1371/journal.pmed.0040118)
Supplement: Supplementary file 1 [file pmed.0040118.sd001.doc]

Resumen

Introducción. La macrosomía comporta una morbilidad maternal y neonatal apreciable. Los factores que predicen la macrosomía son poco conocidos. La elevada frecuencia de macrosomía en hijos de madres diabéticas y en la hiperinsulinemia congénita es debida a un aumento de la secreción de insulina fetal. Hemos valorado el papel intrauterino y neonatal de dos reguladores clave de la secreción de insulina mediante el análisis del peso corporal y la incidencia de hipoglucemia neonatal en sujetos con mutaciones heterocigotas de los genes MODY *HNF4A* (que codifica HNF-4α) y *TCF1* o *HNF1A* (que codifica HNF-1α).

Metodología/Hallazgos Principales. Examinamos el peso al nacer y la existencia de hipoglucemias en 108 sujetos de familias con diabetes por mutaciones *HNF4A*, y en 134 sujetos de familias con mutaciones *HNF1A*. El peso al nacer estaba aumentado en 790 g (p<0,001) en portadores de mutaciones *HNF4A* (50% macrosómicos), en comparación con los familiares que no eran portadores del a mutación (11% macrosómicos, p<0,001). Se detectó hipoglucemia transitoria en 8/54 lactantes con mutaciones *HNF4A*, pero sólo en 0/54 no portadores (p=0,003). Se documentó hiperinsulinemia en tres casos. Los ratones con inactivación en células β del gen *Hnf4α* tenían hiperinsulinemia *in utero* e hipoglucemia hiperinsulinémica al nacer. El peso al nacer y la prevalencia de hipoglucemia neonatal no estaban aumentados en los portadores de mutaciones *HNF1A*.

Conclusiones/significado. Las mutaciones *HNF4A* se asocian a un aumento significativo del peso al nacer y a macrosomía, y son una nueva causa de hipoglucemia. Este estudio establece un papel clave para *HNF4A* como determinante del peso fetal, y descubre una faceta no anticipada de la historia natural de la diabetes por deficiencia de *HNF4A*, en la que existe hiperinsulinemia al nacer que posteriormente evoluciona a una reducción de la secreción de la insulina y diabetes.
